# Palladium-Catalyzed Multicomponent Synthesis of 2-Imidazolines from Imines and Acid Chlorides

**DOI:** 10.3390/molecules171213759

**Published:** 2012-11-22

**Authors:** Boran Xu, Kraig Worrall, Bruce A. Arndtsen

**Affiliations:** Department of Chemistry, McGill University, 801 Sherbrooke Street West, Montreal, QC H3A 2K6, Canada; E-Mails: boran.xu@mail.mcgill.ca (B.X.); kraig.worrall@gmail.com (K.W.)

**Keywords:** 2-imidazoline, palladium, catalysis, multicomponent synthesis

## Abstract

We describe the palladium-catalyzed multicomponent synthesis of 2-imidazolines. This reaction proceeds via the coupling of imines, acid chlorides and carbon monoxide to form imidazolinium carboxylates, followed by a decarboxylation. Decarboxylation in CHCl_3_ is found to result in a mixture of imidazolinium and imidazolium salts. However, the addition of benzoic acid suppresses aromatization, and generates the *trans*-disubstituted imidazolines in good yield. Combining this reaction with subsequent nitrogen deprotection provides an overall synthesis of imidazolines from multiple available building blocks.

## 1. Introduction

2-Imidazolines, the 4,5-reduced counterpart of imidazoles, are an important class of small molecules. The skeletal core of imidazolines can be found in many biological active compounds, including anti-cancer agents [1,2], antidepressants [3,4], analgesics [5,6], and anti-inflammatory agents [7,8]. In addition, imidazolines are useful in coordination chemistry, serving as *N*-donor ligands in various transition metals complexes [9,10,11] or as precursors to *N-*heterocyclic carbenes [12,13]. 

The traditional method to synthesize imidazolines involves the coupling of a 1,2-diamine with an appropriate condensation partner, such as aldehydes, esters, amides or imidates [14,15,16,17,18]. More recently, other synthetic routes to imidazolines have been reported, including the ring opening of aziridines [19,20], the reaction of imines with isocyanates [21,22,23], and the cycloaddition of imines to azomethine ylides [24]. While these methods are each effective, they often require the use of synthetic precursors which can themselves require a multistep synthesis. This can make it challenging to both generate polysubstituted imidazolines, and to diversify their structure to modify properties. 

**Scheme 1:**
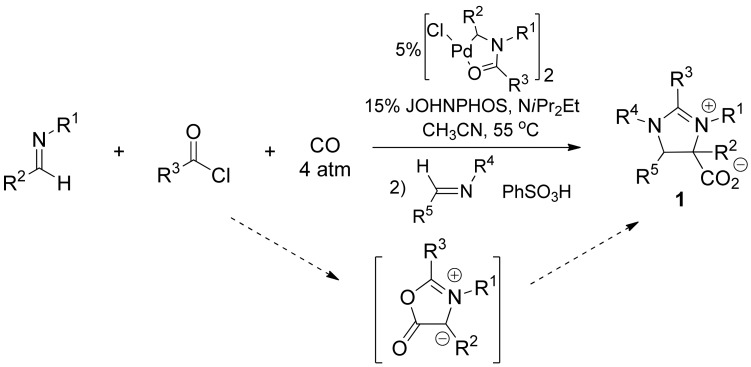
Palladium catalyzed synthesis of imidazolinium carboxylates.

We have previously reported a palladium-catalyzed multicomponent synthesis of imidazolinium carboxylates from imines, acid chlorides and CO (Scheme 1) [25,26,27]. Transition-metal catalyzed multicomponent reactions have emerged as a powerful tool in synthesis as they can allow the controlled assembly of multiple simple units directly into complex products [28,29,30,31,32,33]. The generation of **1** proceeds via imine cycloaddition to an *in situ* generated münchnone (Scheme 1). This reactivity is similar to alkene cycloaddition to münchnones, which, upon themolysis, can undergo decarboxylation to yield pyrrolines [34,35]. As such, we became interested in the analogous reaction of **1**. The latter could provide a modular assembly of imidazolines. Tepe and co-workers have recently reported that similar 2-imidazoline carboxylates can undergo thermal decarboxylation under certain conditions [36]. This observation prompted us to investigate decarboxylation as a platform to access substituted imidazolines from **1**.

## 2. Results and Discussion

**Scheme 2:**
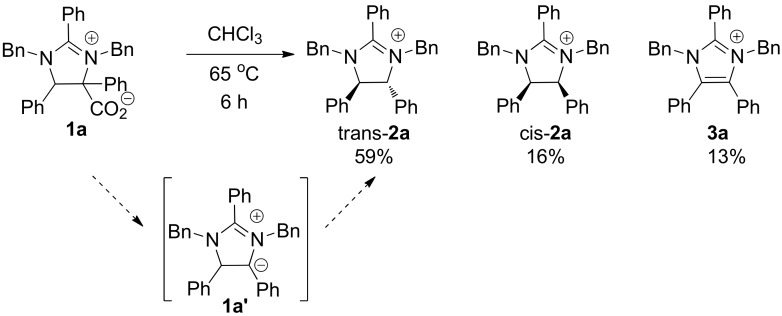
Thermal decarboxylation of imidazolinium carboxylates.

In order to test the viability of this chemistry in imidazoline synthesis, imidazolinium carboxylate **1a** (R^1^, R^4^ = benzyl, R^2^, R^3^, R^5^ = phenyl) was prepared via the palladium catalyzed multicomponent synthesis shown in Scheme 1 [JOHNPHOS = P(t-Bu)_2_(2-biphenyl)]. The heating of **1a** in CDCl_3_ at 65 °C for 6 h leads to the complete conversion of starting compound and the formation of three products, identified as the *trans*- and *cis*-isomers of the imidazolinium **2a**, as well as the aromatized imidazolium **3a** in yields of 59:16:13, respectively (Scheme 2) [37]. The thermal decarboxylation of **1a** presumably proceeds via the initial loss of CO_2_ to generate the ylide **1a'**, which could either be protonated by the solvent (compound **2a**) or undergo oxidation to form **3a**. To favor the generation of **2a**, we examined the influence of acids on the reaction. As shown in Table 1, performing the decarboxylation with one equivalent of benzoic acid leads to the exclusive formation of the imidazoline in 78% yield (entry 1). Other organic carboxylic acids are also effective, and result in similar yields and selectivities (entries 2–3). 

In all cases, no significant amount of aromatized product **3a** is observed, indicating a rapid protonation of the ylide intermediate. The use of stronger acids did not lead to decarboxylation (entries 4–6). In addition, the use of a large excess of benzoic acid inhibited the reaction (entry 7). This suggests that the decarboxylation occurs from zwitterionic **1a**.

The above reactions all generate imidazolium salt **2a** as a mixture of stereoisomers. However, during our studies on this decarboxylation, we were surprised to find that the use of wet chloroform solvent significantly favored the generation of *trans*-**2a** over the *cis*-isomer. This can be performed in a more controlled fashion, where the addition of an excess of water (20 equiv.) with benzoic acid leads to the formation of *trans*-**2a** in 86% yield, and almost completely suppresses the *cis*-product (entry 8). The mechanism by which water influences the protonation is not known, although a control experiment using water without acid leads to decomposition of the starting material (entry 9), suggesting water in concert with benzoic acid results in a proton source that favors proton transfer on the same face as the pendant aromatic unit. 

**Scheme 3:**
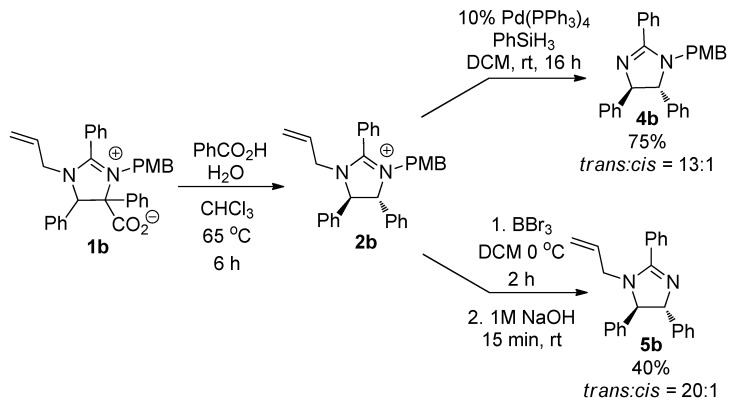
Nitrogen deprotection of imidazolines.

This sequence of palladium catalyzed multicomponent coupling and decarboxylation provides a method to selectively generate *trans*-substituted imidazolinium salts. As imines are readily available from aldehydes and amines, it is straightforward to synthesize asymmetrically substituted imidazolines. This can include the incorporation of orthogonal nitrogen protecting groups. For example, imidazolinium carboxylate **1b** bearing both *N*-allyl and *N-para*-methoxybenzyl (PMB) protecting groups can be generated through palladium catalysis (Scheme 3). Subsequent decarboxylation followed by deallylation yields imidazoline **4b**. Alternatively, the *para*-methoxybenzyl group can be cleaved from imidazolinium cation **2b**, affording imidazoline **5b**.

**Scheme 4:**
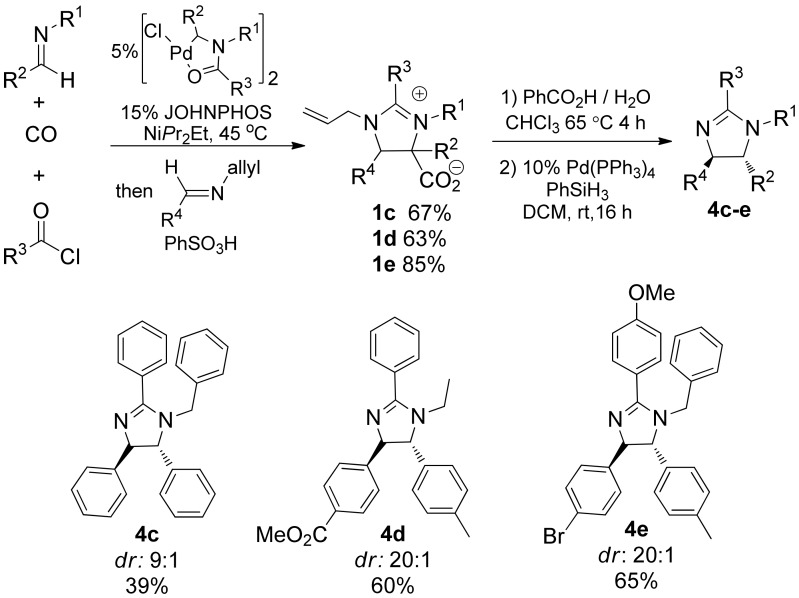
Modular synthesis of imidazolines.

As an illustration of the versatility of this approach, a number of orthogonally substituted imidazolines have been generated via the palladium catalyzed synthesis of **1** and selective decarboxylation (Scheme 4). The substituents of the imidazoline core can be varied by choosing the appropriate imine(s) and acid chloride, while functional groups such as esters, aryl ethers, alkenes and aryl-halides are all tolerated. This modularity can allow for the rapid synthesis of imidazolines with independent control of four separate substituents.

## 3. Experimental

### 3.1. General Considerations

All solvents used were dried by passage through a column of alumina prior to use. All common reagents were purchased from Aldrich (Oakville, Canada) and used as received unless otherwise noted. Pd(PPh_3_)_4_ was purchased from Strem (Boston, MA, USA) and stored in a nitrogen glovebox. Imines were synthesized by the condensation of the appropriate aldehyde and amine in the presence of MgSO_4_ and purified by distillation under vaccum, according literature methods [38]. Amide-chelated palladium catalysts were synthesized according to a literature protocol [39]. *N,N*-diisopropylethylamine was distilled over CaH_2_ prior to use. Carbon monoxide was purchased at 99.99% purity MEGS (Montreal, Canada) and used as received. NMR characterization was performed at 300 MHz, 400 MHz and 500 MHz for ^1^H-NMR and 75 MHz and 126 MHz for ^13^C-NMR on Varian spectrometers. Resonances at 145.5 and 53.2 ppm in the ^13^C-NMR spectra are artifacts generated during data collection and do not represent real product signals. Chemical shifts are reported in parts per million relative to the residual solvent signal. Mass spectra were recorded on a Agilent LC-MSD TOF high-resolution electrospray ionization quadrupole spectrometer. 

### 3.2. Synthesis of trans-1,3-Dibenzyl-2,4,5-triphenyl-4,5-dihydro-3H-imidazol-1-ium (trans-***2a***) and cis-1,3-Dibenzyl-2,4,5-triphenyl-4,5-dihydro-3H-imidazol-1-ium (cis-***2a***)

Imidazolinium carboxylate **1a** (52.2 mg, 0.100 mmol) and benzoic acid (12.2 mg, 0.100 mmol) were dissolved in CHCl_3_ (5 mL). Benzyl benzoate (10 μL) was added as an internal standard and an aliquot of this mixture was taken for ^1^H-NMR analysis. Water (36 μL, 2 mmol) was added by micropipette and the vial was capped and heated at 65 °C for 6 h. The solvent was then removed and the yield of the products was determined by ^1^H-NMR analysis in CDCl_3_. The identity of *cis*- and *trans*-**2a** was determined by the palladium-catalyzed cleavage of the benzyl protecting groups (5 mol% Pd(OH)_2_, AcOH, 65 °C, 48 h), and comparing the resulting imidazoline to previously reported compounds [16,40].

### 3.3. Synthesis of 1,3-Dibenzyl-2,4,5-triphenyl-3H-imidazol-1-ium *(**3a**)*

In a septum sealed NMR tube, 1-benzyl-2,4,5-triphenyl-1*H*-imidazole [41] (19.5 mg, 0.05 mmol) was dissolved in CDCl_3_ (1.5 mL). Benzyl bromide (9.0 μL, 0.075 mmol) was added by microsyringe. The tube was heated at 75 °C for 3 days. The solvent was removed *in vacuo* and the resulting residue was redissolved in CH_2_Cl_2_ (500 μL). Diethyl ether (10 mL) was slowly layered on top of this solution, resulting in the precipitation of a white solid. This solid was further triturated with diethyl ether to afford **3a** as a white solid. (12.2 mg, 43%) ^1^H-NMR (500 MHz, CDCl_3_): δ 8.00–7.99 (m, 1H), 7.56–7.53 (m, 3H), 7.46 (t, *J* = 7.7 Hz, 1H), 7.32–7.29 (m, 1H), 7.27–7.23 (m, 2H), 7.20–7.14 (m, 3H), 6.76–6.75 (m, 2H), 5.25 (s, 2H). ^13^C-NMR (126 MHz, CDCl_3_): δ 145.1, 134.1, 132.8, 132.3, 131.5, 131.4, 130.0, 129.4, 128.8, 128.7, 128.2, 127.1, 125.4, 122.1, 50.7 HRMS (ESI): C_35_H_29_N_2_ (M)^+^ calcd; 477.23359 observed; 477.23253

### 3.4. Synthesis of 1-(4-Methoxy-benzyl)-2,4,5-triphenyl-4,5-dihydro-1H-imidazole *(**4b**)*

Compound **1b** was prepared according to a literature procedure [25]. In a glovebox under nitrogen atmosphere, (4-CH_3_C_6_H_4_)HC=NCH_2_(4-C_6_H_4_OCH_3_) (67.8 mg, 0.300 mmol) and benzoyl chloride (54.8 mg, 0.390 mmol) were dissolved in CH_3_CN (5 mL). The mixture was then transferred to a 50 mL thick walled Schlenk tube and allowed to stir for 15 min. [Pd(Cl)[η^2^-CH(4-CH_3_C_6_H_4_)-NCH_2_(4-C_6_H_4_OMe)COPh]]_2_ (14.2 mg, 0.015 mmol), P(*t*-Bu)_2_(2-biphenyl) (13.4 mg, 0.045 mmol) and *N,N*-diisopropylethylamine (50.3 mg, 0.390 mmol) were combined in THF (5 mL) and added to the mixture. The solution was stirred for 5 min before the Schlenk tube was briefly evacuated, brought outside of the glovebox, charged with 4 atm of CO, and heated at 45 °C for 16 h. The CO atmosphere was replaced with nitrogen, the tube was brought into the glovebox, and PhHC=N(CH_2_CH=CH_2_) (87.0 mg, 0.600 mmol) and PhSO_3_H (40.0 mg, 0.25 mmol) in THF (2 mL) were added. The mixture was stirred for 16 h. The reaction mixture was diluted with CH_2_Cl_2_ (25 mL), then sequentially washed with brine, 0.1 M HCl and sat. NaHCO_3_, extracting with additional CH_2_Cl_2_ in each wash. The combined organic layers were dried with Na_2_SO_4_, filtered and concentrated to 1 mL of solvent. Diethyl ether (20 mL) was slowly layered on the solution resulting in precipitation of the product, which was subsequently triturated with additional diethyl ether, providing **1b** as a yellow solid which was immediately used the subsequent decarboxylation reaction. 

To a sample of **1b** (50.2 mg, 0.100 mmol), was added benzoic acid (12.2 mg, 0.100 mmol) in CHCl_3_ (5 mL). Water (36 μL, 2 mmol) was added by micropipette and the vial was capped and heated at 65 °C for 6 h. The mixture was allowed to cool to room temperature, diluted with CH_2_Cl_2_ (20 mL), then washed with brine, extracting with additional CH_2_Cl_2_. The combined organic layers were dried with Na_2_SO^−^_4_, filtered and concentrated to give imidazolinium **2b** as a yellow solid. This compound was transferred without further purification to a dry Schlenk flask and placed under N_2_. Pd(PPh_3_)_4_ (11.5 mg, 0.010 mmol) was added as a solution in dry CH_2_Cl_2_ (2 mL) followed by PhSiH_3_ (25.0 μL, 0.2 mmol) in CH_2_Cl_2_ (2 mL). The reaction mixture was allowed to stir at room temperature for 16 h. The solution was then diluted with additional with CH_2_Cl_2_ (20 mL) and washed with brine, extracting with additional CH_2_Cl_2_. The organic layers were dried with Na_2_SO^−^_4_, filtered and concentrated to give a brown solid which was purified by flash column chromatography (5% MeOH in CH_2_Cl_2_) to yield imidazoline **4b** as a brown solid (31.9 mg, 76% *dr*: 13:1) ^1^H-NMR (300 MHz, CDCl_3_): δ 7.84 (dt, *J* = 6.7, 3.3 Hz, 2H), 7.57–7.51 (m, 4H), 7.43–7.20 (m, 10H), 7.12–7.07 (m, 2H), 6.92–6.83 (m, 2H), 6.75 (d, *J* = 8.6 Hz, 2H), 5.03–5.00 (m, 1H), 4.70 (d, *J* = 15.3 Hz, 1H), 4.38–4.35 (m, 1H), 3.87 (d, *J* = 15.3 Hz, 1H), 3.76 (d, *J* = 7.0 Hz, 3H). ^13^C-NMR (75 MHz; CDCl_3_): δ 166.0, 159.0, 143.3, 141.3, 130.5, 129.3, 128.9, 128.8_3_, 128.7_7_, 128.6_9_, 128.5, 127.9_7_, 127.9_0_, 127.8_8_, 127.2, 126.7, 113.9, 77.2, 72.3, 55.3, 49.0 HRMS (ESI): C_29_H_26_N_2_O (M+H)^+^ calcd; 419.21179 observed; 419.21203.

*1-Benzyl-2,4,5-triphenyl-4,5-dihydro-1H-imidazole* (**4c**). Yield: 39% *dr*: 9:1); ^1^H-NMR (400 MHz, CDCl_3_): δ 7.86–7.81 (m, 2H), 7.51–7.48 (m, 3H), 7.41–7.19 (m, 12H), 7.13–7.11 (m, 2H), 6.97–6.94 (m, 2H), 5.00 (d, *J* = 8.6 Hz, 1H), 4.73 (d, *J* = 15.6 Hz, 1H), 4.35 (d, *J* = 8.6 Hz, 1H), 3.93 (d, *J* = 15.6 Hz, 1H). ^13^C-NMR (75 MHz, CDCl_3_): δ 166.0, 143.8, 141.8, 136.4, 131.2, 130.2, 128.9, 128.7_2_, 128.6_8_, 128.5, 128.4, 128.0, 127.8, 127.5, 127.2, 127.0, 126.8, 77.2, 72.5, 49.6 HRMS (ESI): C_28_H_24_N_2_ (M+H)^+^ calcd; 389.20123 observed; 389.20148.

*4-(1-Ethyl-2-phenyl-5-p-tolyl-4,5-dihydro-1H-imidazol-4-yl)-benzoic acid methyl ester* (**4d**). Yield: 60% *dr*: 20:1; ^1^H-NMR (500 MHz, CDCl_3_) δ 8.04–8.03 (m, 2H), 7.91–7.89 (m, 1H), 7.79–7.76 (m, 2H), 7.53–7.47 (m, 3H), 7.42–7.38 (m, 3H), 7.32–7.22 (m, 5H), 5.15 (d, *J* = 8.9 Hz, 1H), 4.46 (d, *J* = 9.0 Hz, 1H), 3.35–3.28 (m, 1H), 3.11–3.03 (m, 1H), 2.39 (s, 3H), 0.90–0.84 (m, 4H). ^13^C-NMR (CDCl_3_, 126 MHz): δ 167.2, 167.0, 148.5, 138.2, 138.1, 130.6, 130.0, 129.8, 129.2, 128.6, 128.4, 127.8, 127.1, 126.6, 75.9, 73.9, 52.1, 41.1, 21.2, 13.1 HRMS (ESI): C_26_H_26_N_2_O_2_ (M+H)^+^ calcd; 399.20670 observed; 399.20664.

*1-Benzyl-4-(4-bromo-phenyl)-2-(4-methoxy-phenyl)-5-p-tolyl-4,5-dihydro-1H-imidazole* (**4e**). Yield 63% *dr*: 20:1; ^1^H-NMR (500 MHz, CDCl_3_): δ 7.77–7.74 (m, 2H), 7.37–7.34 (m, 2H), 7.24–7.16 (m, 7H), 7.02–6.99 (m, 2H), 6.96–6.92 (m, 4H), 4.91 (d, *J* = 8.6 Hz, 1H), 4.75 (d, *J* = 15.6 Hz, 1H), 4.21 (d, *J* = 8.7 Hz, 1H), 3.88 (d, *J* = 15.6 Hz, 1H), 3.87(s, 3H), 2.39 (s, 3H). ^13^C-NMR (75 MHz, CDCl_3_): δ 166.0, 161.3, 142.8, 138.1, 137.9, 136.2, 131.5, 130.3, 129.7, 128.6, 128.4, 128.0, 127.7, 127.1, 122.6, 121.0, 114.1, 76.3, 72.2, 55.4, 49.5, 21.2 HRMS (ESI): C_30_H_27_BrNO_2_ (M+H)^+^ calcd; 511.13795 observed; 511.13808.

### 3.5. Synthesis of 1-Allyl-2,4,5-triphenyl-4,5-dihydro-1H-imidazole *(**5b**)*

Compound **2b** (95.6 mg, 0.19 mmol) was prepared as described above, and transferred without further purification to a dry Schlenk flask and placed under N_2_. The solid was dissolved in dry CH_2_Cl_2_ (5 mL) and cooled to 0 °C. BBr_3_ (1.0 M in CH_2_Cl_2_, 2 mL, 2 mmol, 10 equiv.) was added slowly and the mixture was allowed to warm to room temperature. The solution was stirred at room temperature for 2 h. Sodium hydroxide (10 mL 1.0M solution) was rapidly added to the solution, which was then diluted with CH_2_Cl_2_ (20 mL), and washed with brine, extracting with additional CH_2_Cl_2_. The organic layers were dried with Na_2_SO^−^_4_, filtered, and concentrated to give a yellow oil which was purified by flash column chromatography (5% MeOH in CH_2_Cl_2_) to give a imidazoline **5b** as a yellow oil (27.8 mg, 40%, *dr*: 20:1) ^1^H-NMR (400 MHz, CDCl_3_): δ 7.78–7.74 (m, 2H), 7.51–7.44 (m, 3H), 7.43–7.32 (m, 6H), 7.30–7.26 (m, 3H), 5.55 (m, 1H), 5.06–4.92 (m, 3H), 4.51 (d, *J* = 8.9 Hz, 1H), 3.96–3.91 (m, 1H), 3.47 (dd, *J* = 16.1, 7.3 Hz, 1H). ^13^C-NMR (126 MHz; CDCl_3_): δ 166.4, 144.0, 142.0, 133.0, 131.2, 130.1, 128.9, 128.5_2_, 128.4_7_, 128.4_5_, 127.8, 127.2, 127.1, 126.8, 118.2, 77.8, 74.0, 49.1 HRMS (ESI): C_24_H_22_N_2_ (M+H)^+^ calcd; 339.18558 observed; 339.18605.

## 4. Conclusions

In conclusion, the coupling of the palladium catalyzed multicomponent synthesis of imidazolium carboxylates with stereoselective decarboxylation provides a new and modular synthesis of imidazolines. Efforts towards elucidating the mechanism of the decarboxylation reaction, and directing this towards other classes of heterocyclic products, are currently underway.

## Figures and Tables

**Table 1:**
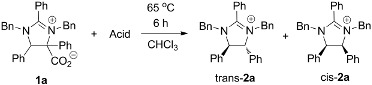
Influence of acids on the generation of imidazolinium salts.

Entry	Acid	Yield 2a	*trans* : *cis* 2a
1	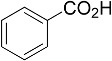	78%	3.2:1
2	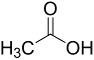	78%	2.7:1
3	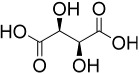	87%	2.7:1
4	HCl	- ^a^	-
5	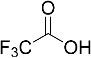	- ^a^	-
6	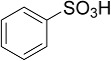	- ^a^	-
7	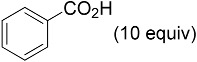	24%	1.5:1
8	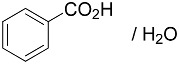	86%	20:1
9	H_2_O (20 equiv.)	- ^b^	-

*Reaction conditions*: **1a** (0.1 mmol) and acid (0.1 mmol) in 5 mL CHCl_3_ heated at 65 °C for 6 h. Yields determined by ^1^H-NMR relative to a benzyl benzoate internal standard. ^a^ Recovery of protonated starting material. ^b^ Decomposition of starting material.

## Supplementary Materials

Supplementary materials can be accessed at: http://www.mdpi.com/1420-3049/17/12/13759/s1.
