# Supplementary material for: Palladium-Catalyzed Multicomponent Synthesis of 2-Imidazolines from Imines and Acid Chlorides
Source: Molecules. 2012 Nov 22;17(12):13759–68. doi: 10.3390/molecules171213759 (PMC6269033; doi:10.3390/molecules171213759)

Supplementary Information

Figure S1. ^{1}H-NMR of compound **3a**.

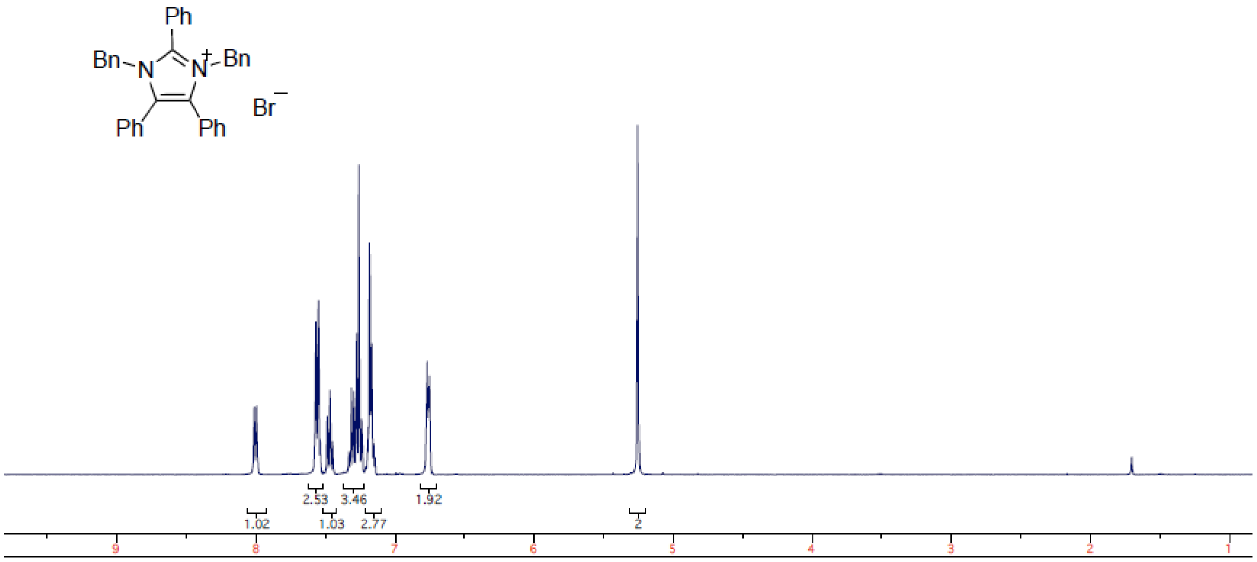

Figure S2. ^{13}C-NMR of compound **3a**.

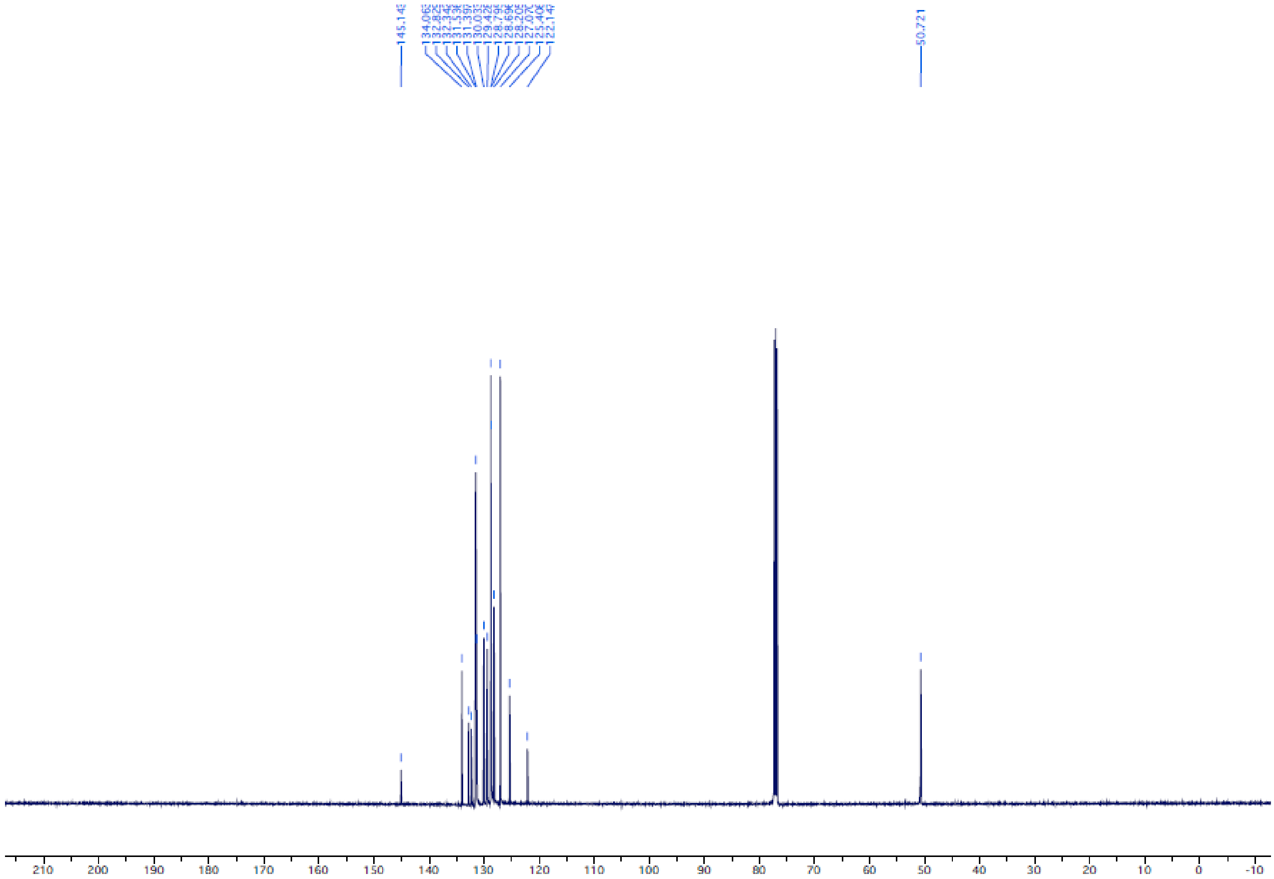

Figure S3. ^{1}H-NMR of compound **4b**.

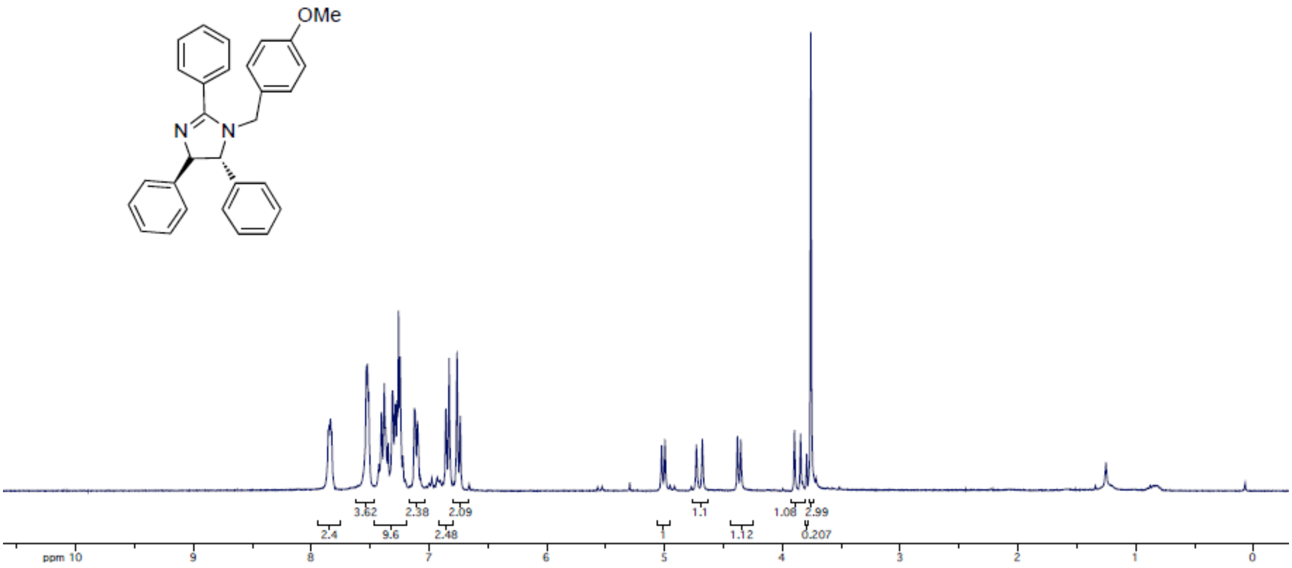

Figure S4. ^{13}C-NMR of compound **4b**.

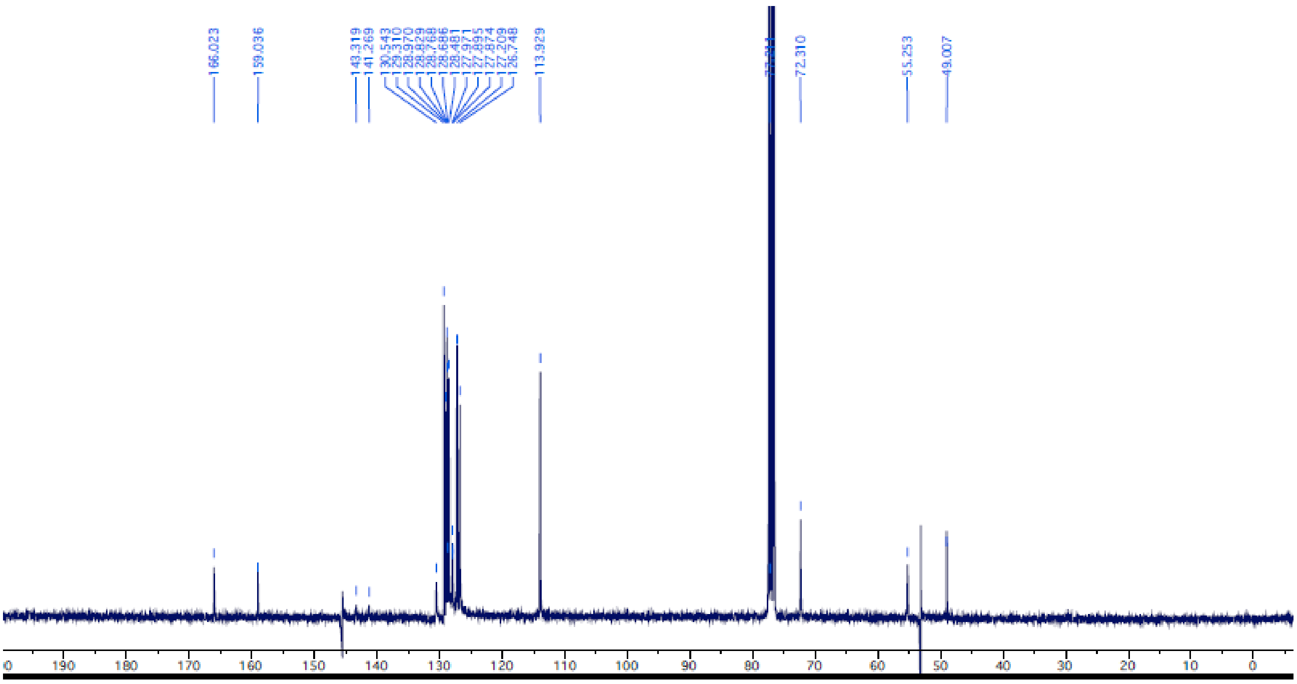

Figure S5. ^{1}H-NMR of compound **5b**.

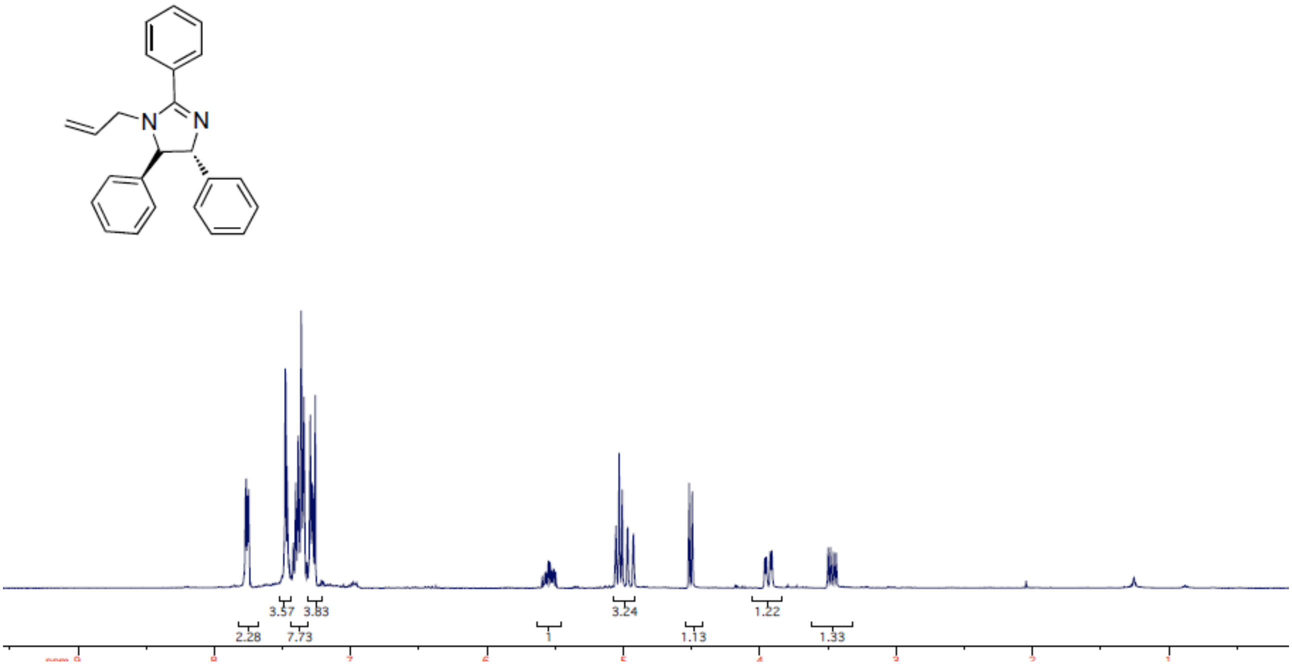

Figure S6. ^{13}C-NMR of compound **5b**.

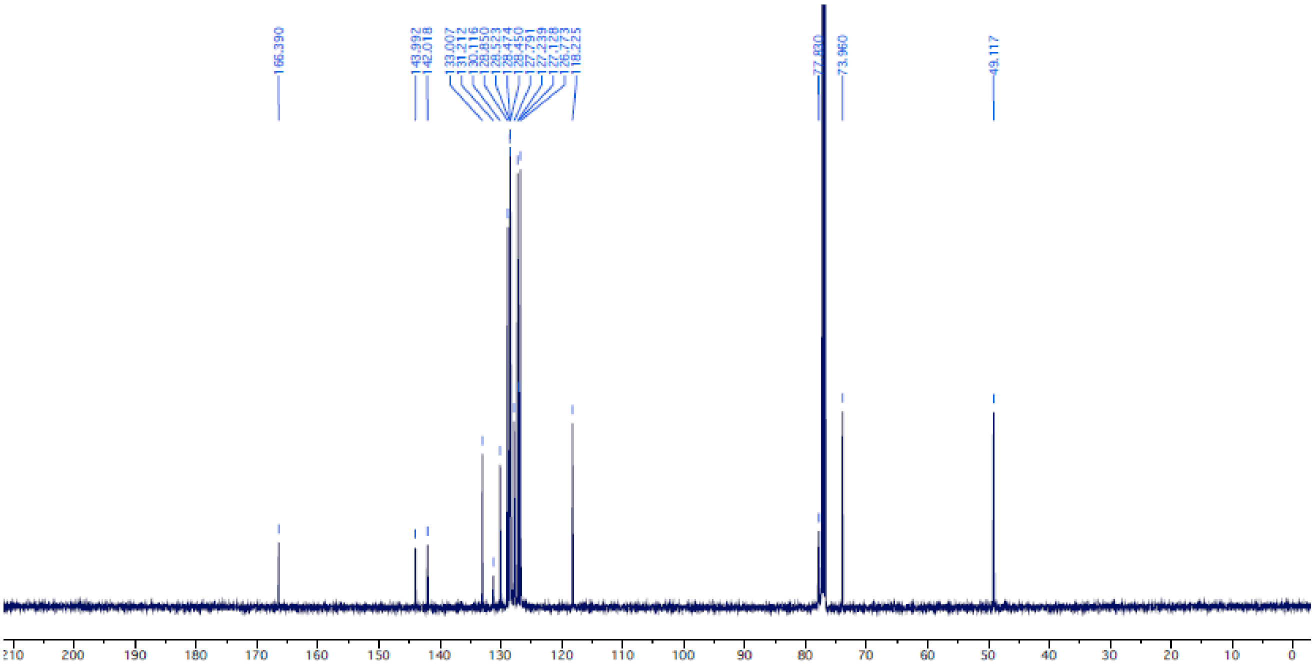

Figure S7. ^{1}H-NMR of compound **4c**.

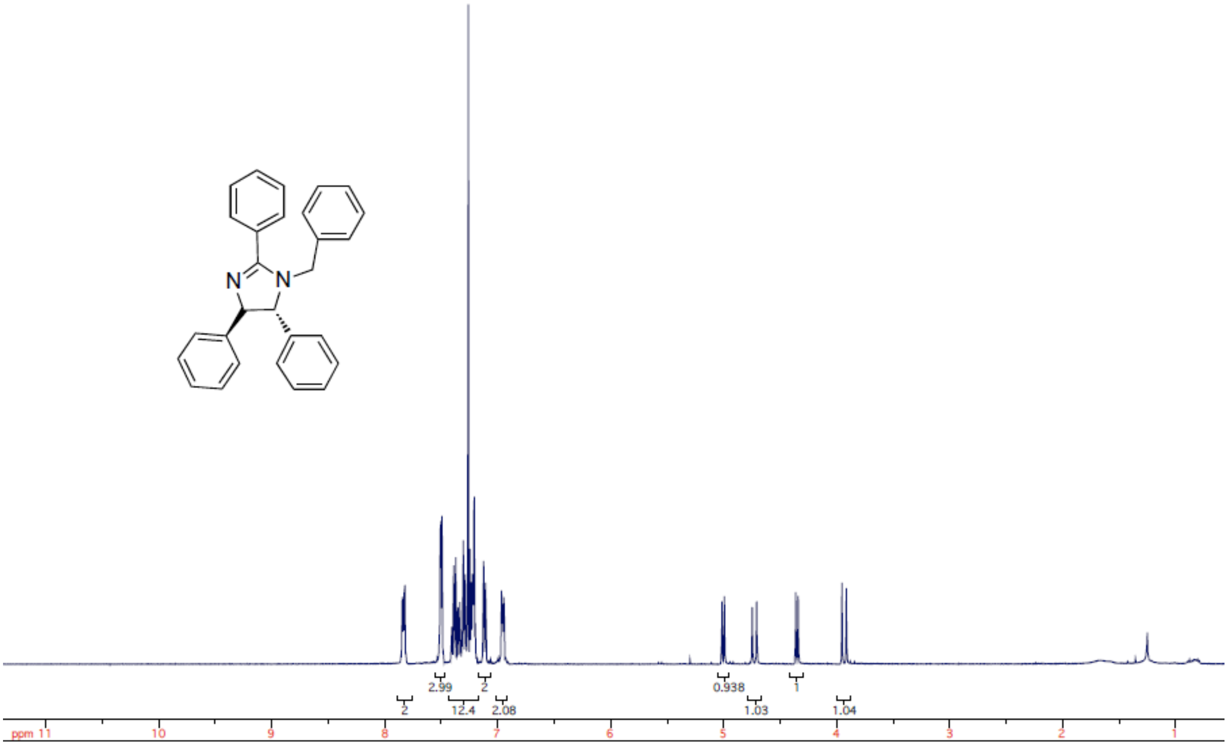

Figure S8. ^{13}C-NMR of compound **4c**.

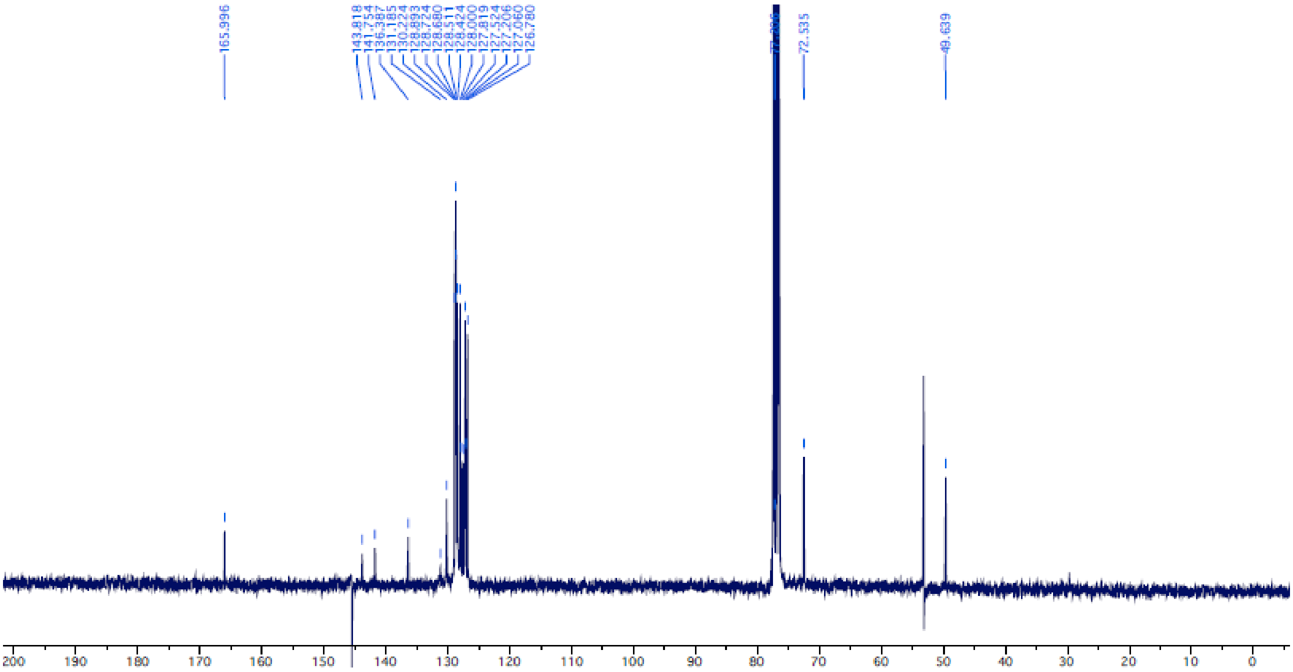

Figure S9. ^{1}H-NMR of compound **4d**.

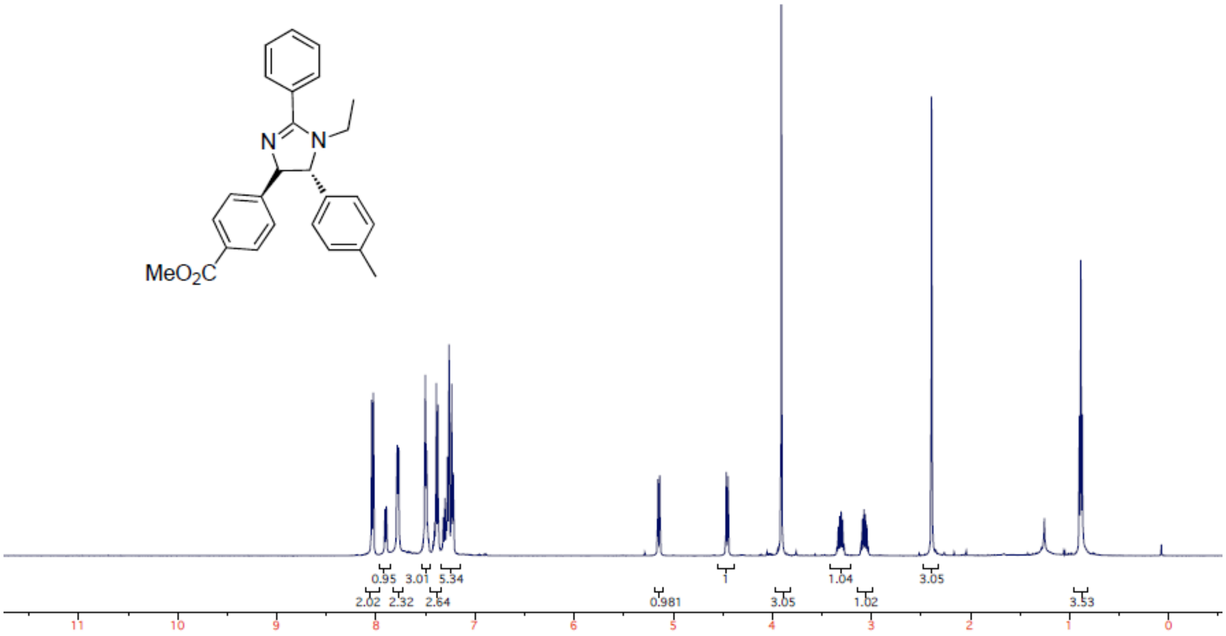

Figure S10. ^{13}C-NMR of compound **4d**.

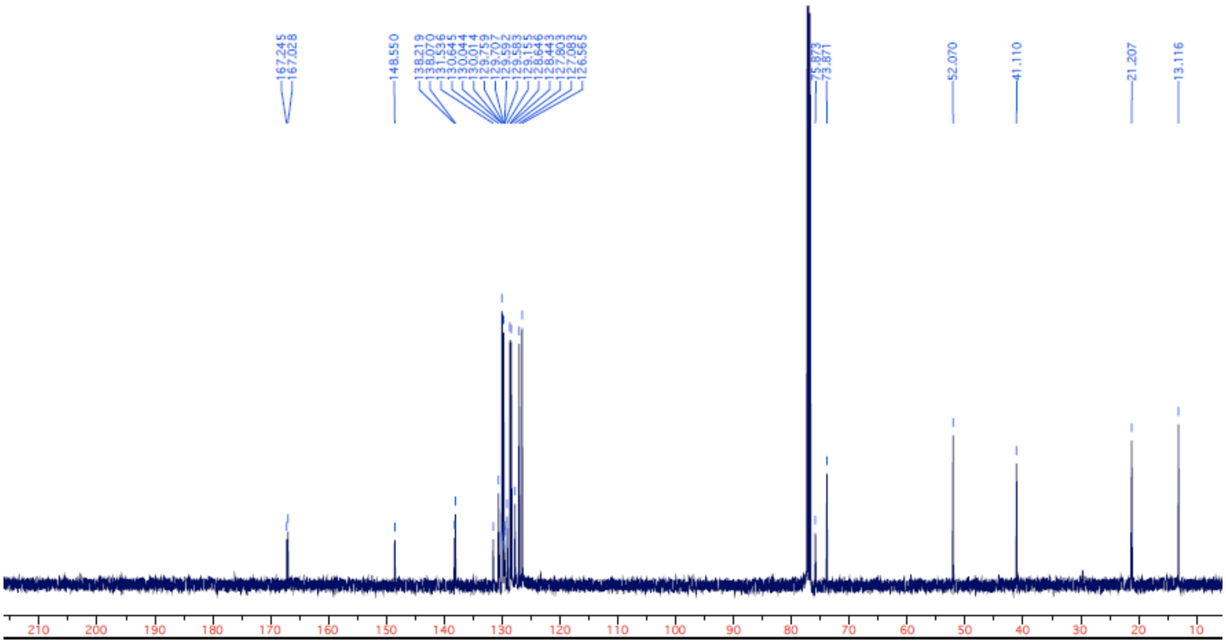

Figure S11. ^{1}H-NMR of compound **4e**.

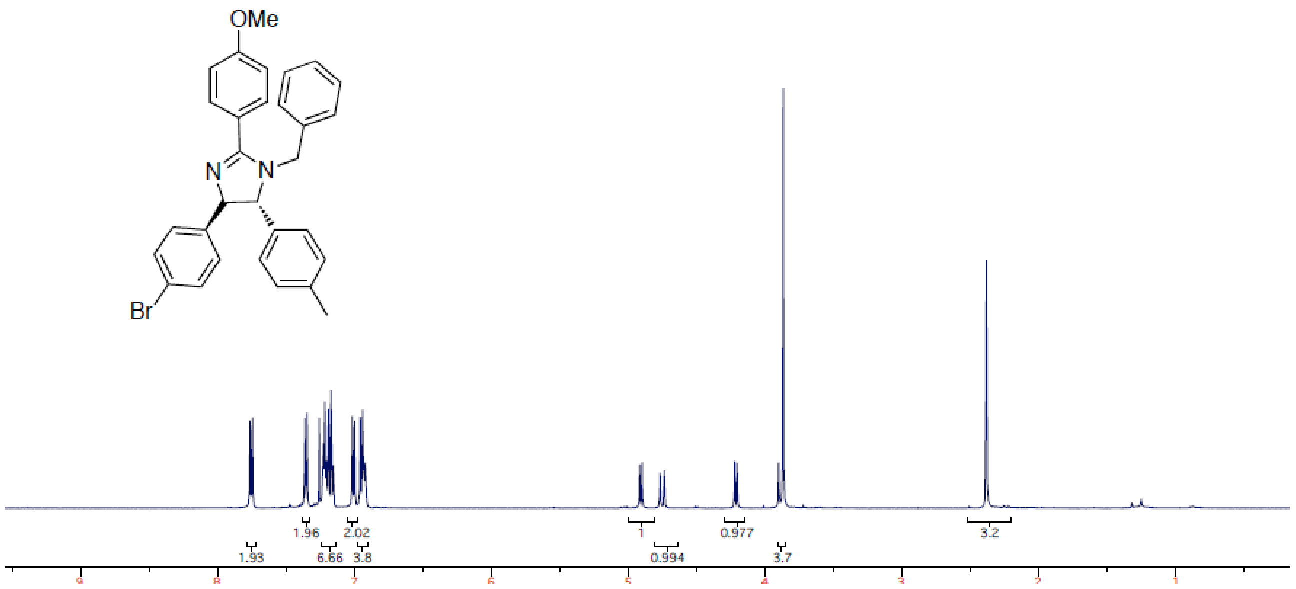

Figure S12. ^{13}C-NMR of compound **4e**.

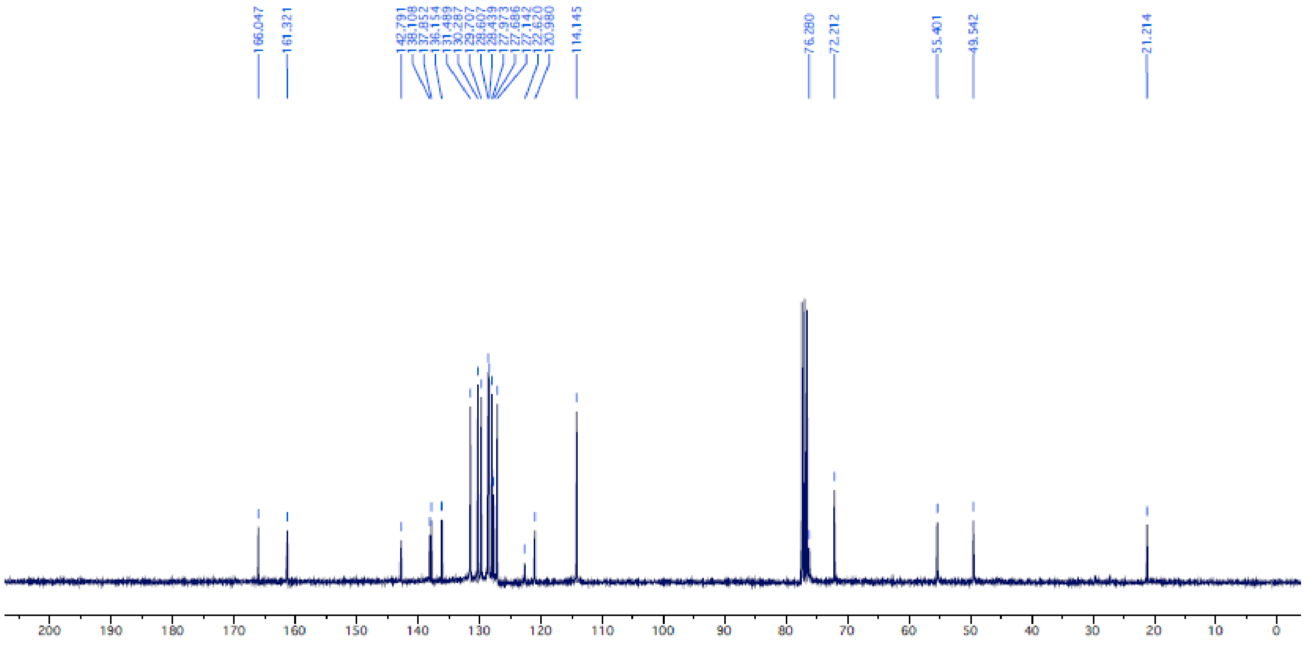

Supplement: Supplementary file 1 [file molecules-17-13759-s001.pdf]
